# Addressing the Gero-Behavioral Health Workforce Shortage: Insights From Oregon

**DOI:** 10.1093/geroni/igaf122.1977

**Published:** 2025-12-31

**Authors:** Lindsey Smith, Brenda Sulick

## Abstract

Older adults experience significant behavioral health (BH) challenges, including substance use disorders (SUD), serious mental illness (SMI), and other conditions such as depression and anxiety. While BH prevalence rates are lower among older adults than younger cohorts, aging-related complexities, comorbidities, and diagnostic overshadowing contribute to severe impacts on health and quality of life. The growing demand for BH services is compounded by systemic barriers, including workforce shortages, stigma, and disparities in access to care—particularly for racially and ethnically diverse populations, rural residents, and those in long-term care settings. This panel will provide a summary of the national-level needs and current efforts before describing the work of the Oregon Center of Excellence for Behavioral Health and Aging (OCEBHA), the first state-level center of excellence in the United States specifically focused on addressing the intersectional needs of older adults with behavioral health needs. Specifically, we will share the structure and funding of OCEBHA, as well as feedback and successes from our inaugural behavioral health and aging conference. A key component of OCEBHA’s work is its partnerships with state agencies, healthcare providers, community-based organizations, and advocacy groups. These collaborations have strengthened our ability to address systemic challenges, promote evidence-based practices, and elevate the voices of older adults and their caregivers. Finally, our discussant will facilitate a conversation about lessons for other states, remaining needs, future directions, and policy implications.

